# Supplementary material for: Lanthanide-regulating Ru-O covalency optimizes acidic oxygen evolution electrocatalysis
Source: Nat Commun. 2024 Jun 11;15:4974. doi: 10.1038/s41467-024-49281-2 (PMC11166638; doi:10.1038/s41467-024-49281-2)
Supplement: Supplementary file 1 — Supplementry Information [file 41467_2024_49281_MOESM1_ESM.pdf]

Supplementary Information

Lanthanide-regulating Ru-O covalency optimizes acidic oxygen evolution electrocatalysis

Li *et al.*

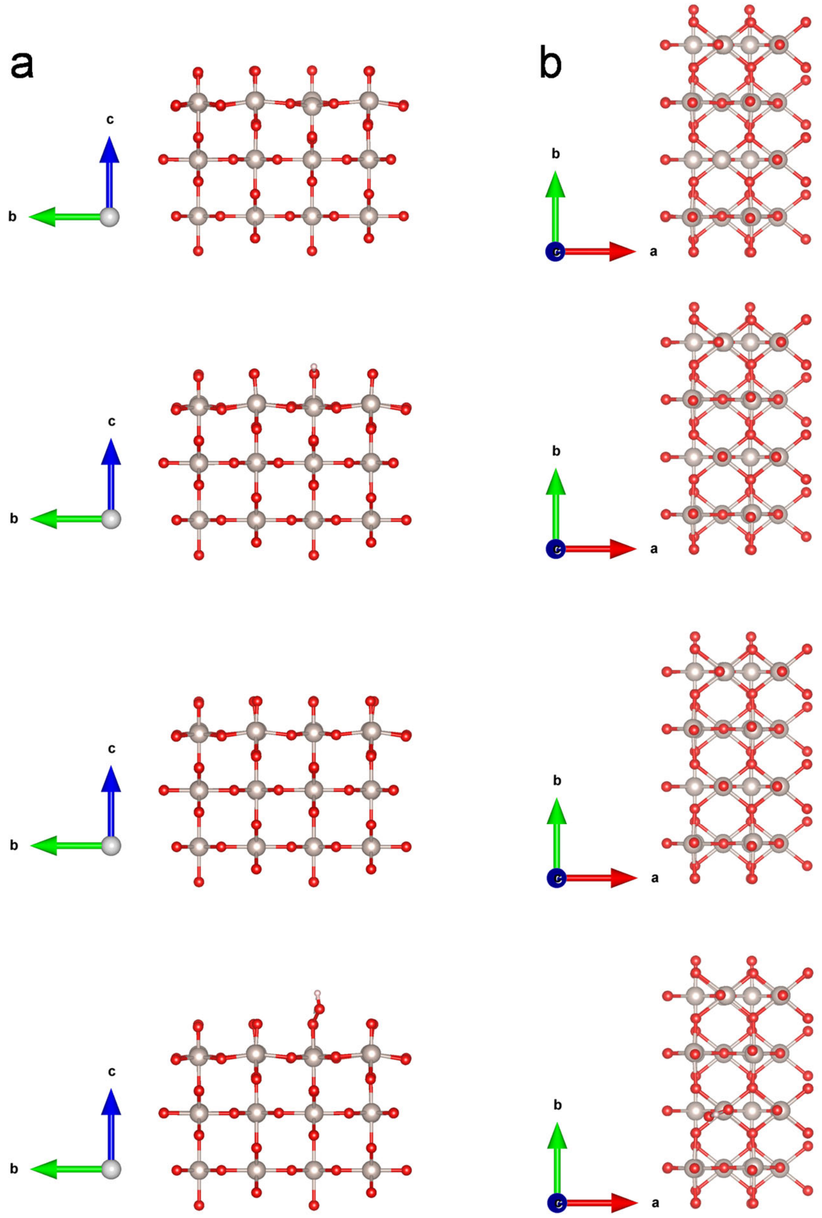

Supplementary Fig. 1| The optimized atomic models for intermediates on RuO_2(110). (a) side view, (b) top view.

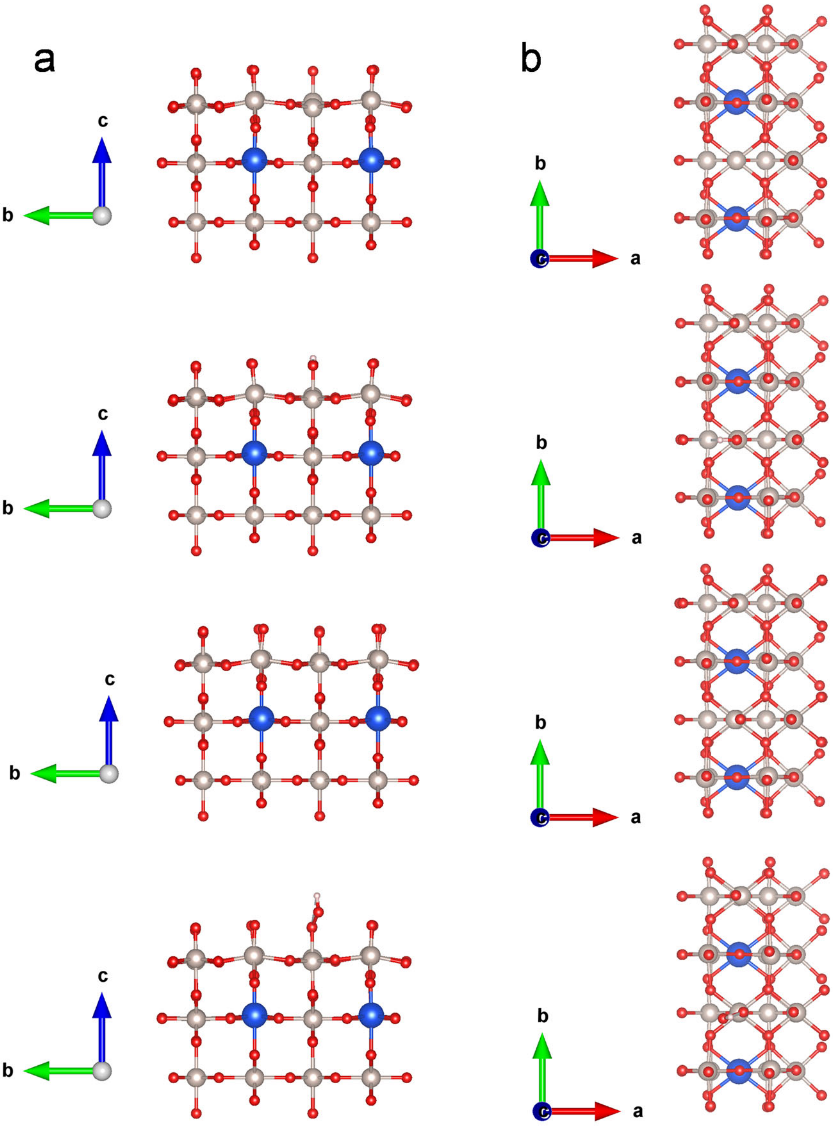

Supplementary Fig. 2| The optimized atomic models for intermediates on Ho-RuO$_x$(110). (a) side view, (b) top view.

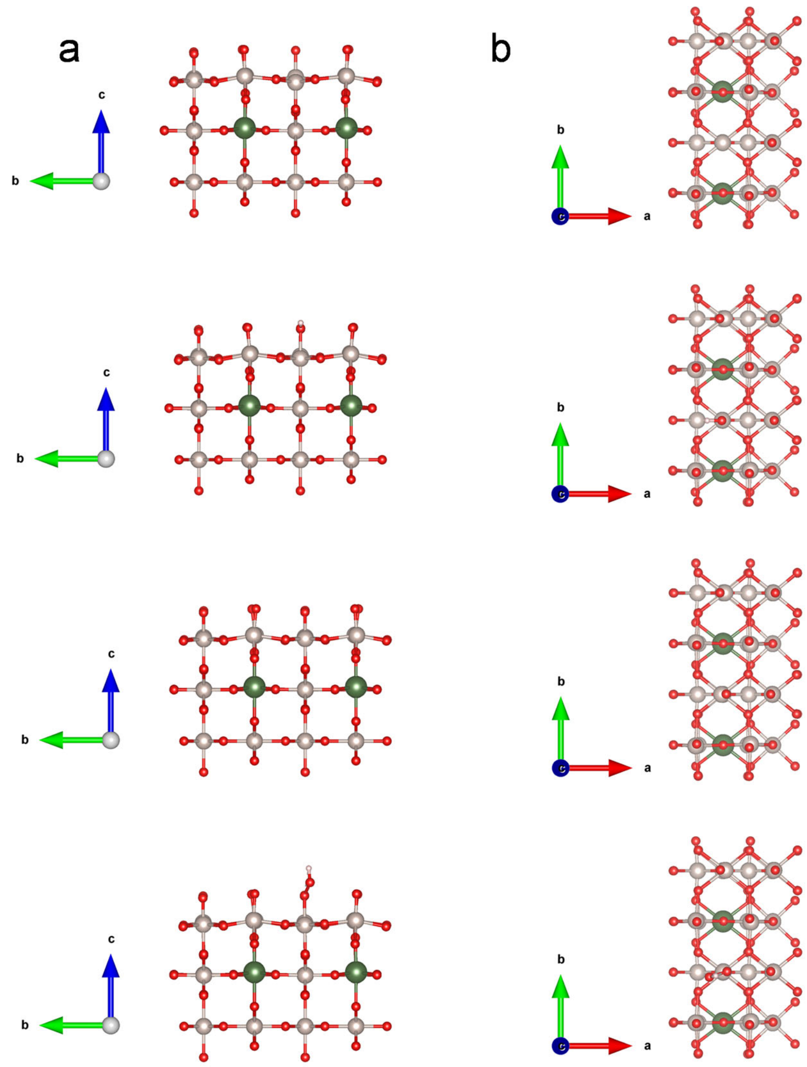

Supplementary Fig. 3| The optimized atomic models for intermediates on Er-RuO$_x$(110). (a) side view, (b) top view.

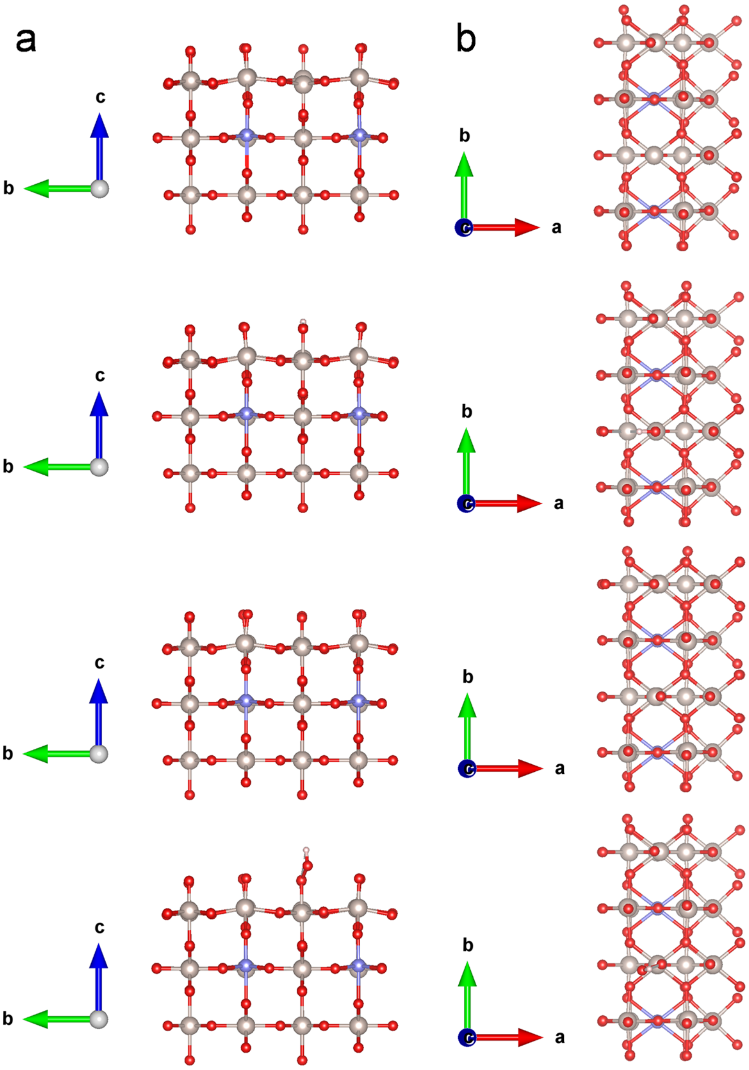

Supplementary Fig. 4| The optimized atomic models for intermediates on Tm-RuO_x(110). (a) side view, (b) top view.

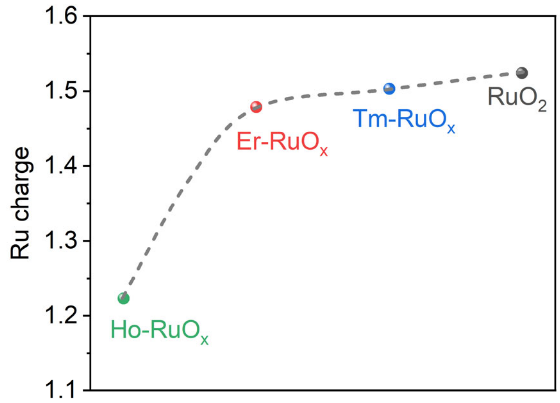

Supplementary Fig. 5| The variation of Ru charge in Ln-RuO$_x$ and RuO$_2$. The calculated Ru charge of Ho-RuO$_x$, Er-RuO$_x$, Tm-RuO$_x$ and RuO$_2$, respectively.

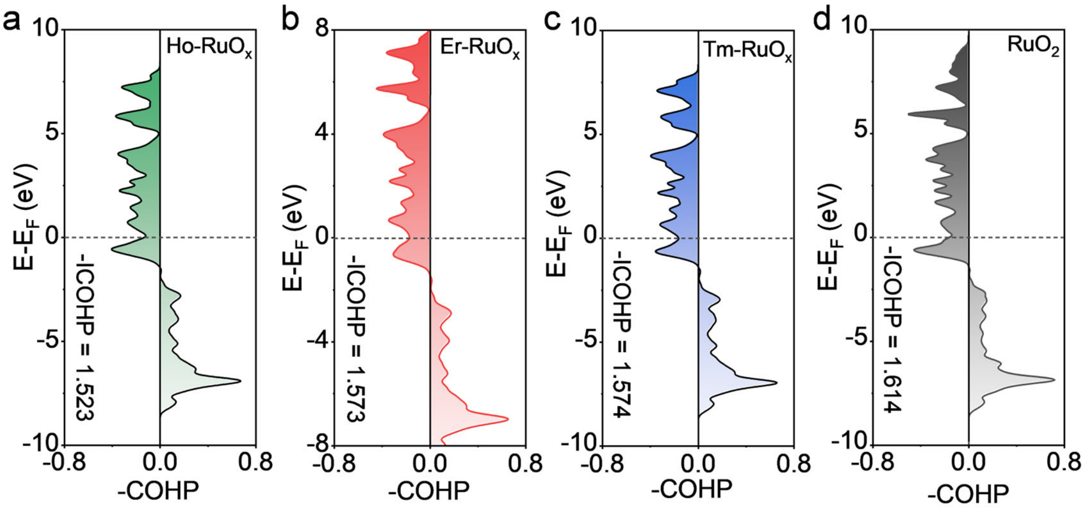

Supplementary Fig. 6| Calculated COHP of the as-prepared catalysts. The COHP diagram of (a) Ho-RuO$_x$, (b) Er-RuO$_x$, (c) Tm-RuO$_x$ and (d) RuO$_2$, respectively.

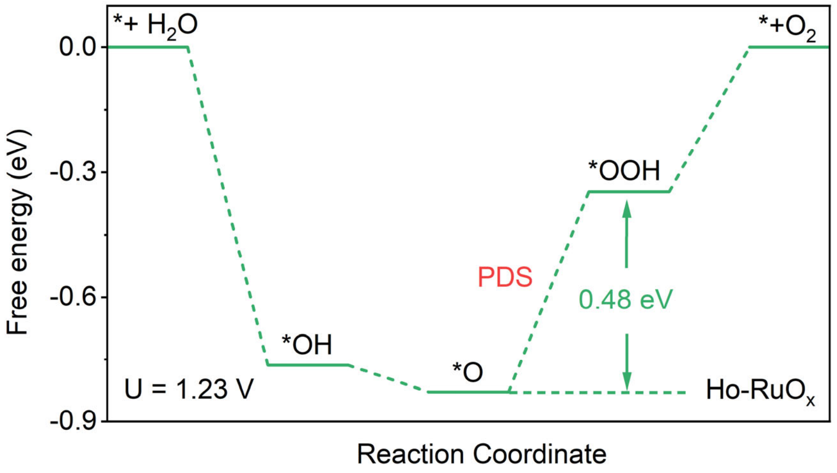

Supplementary Fig. 7| The reaction paths on Ho-RuO_x with the set potential of 1.23 V.

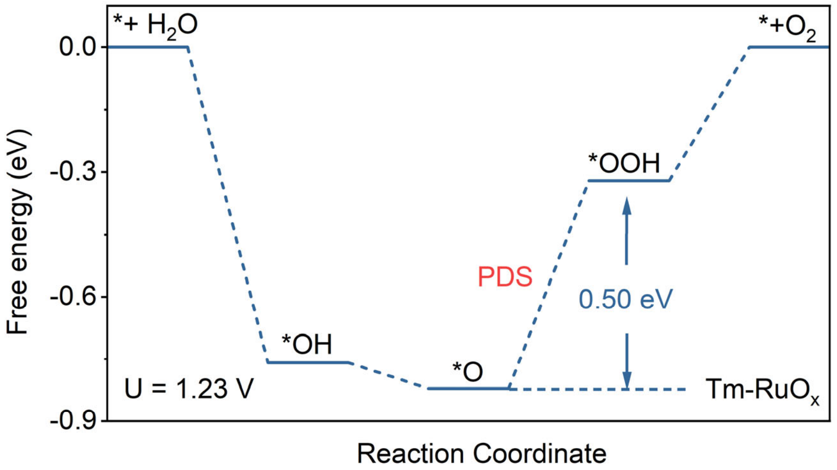

Supplementary Fig. 8| The reaction paths on Tm-RuO_x with the set potential of 1.23 V.

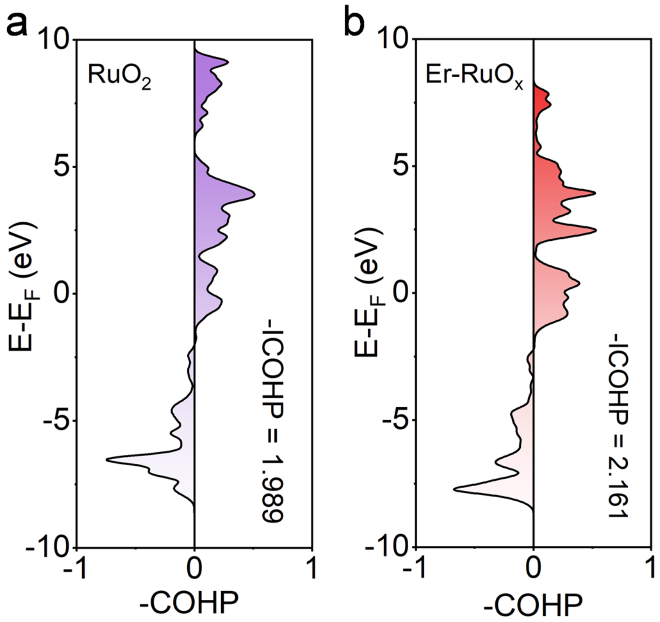

Supplementary Fig. 9| Calculated COHP for Ru-O between *OH and catalysts: (a) RuO_2 and (b) Er-RuO_x.

Supplementary Notes

COHP calculations (Supplementary Fig. 9) demonstrated that introduction of Er could obviously increase the Ru-*OH bonding state occupancy, contributing to a higher OER activity.

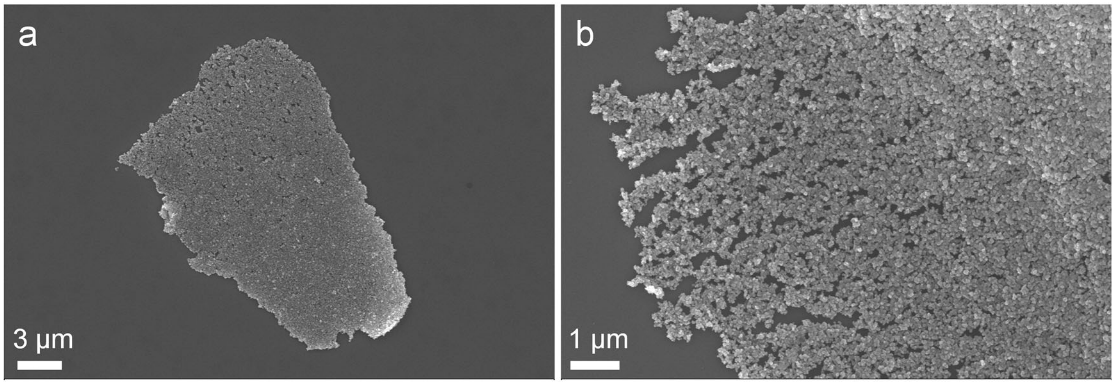

Supplementary Fig. 10| SEM characterization of Er-RuO$_x$. SEM images at various magnification showing the sheet-like porous structures of Er-RuO$_x$.

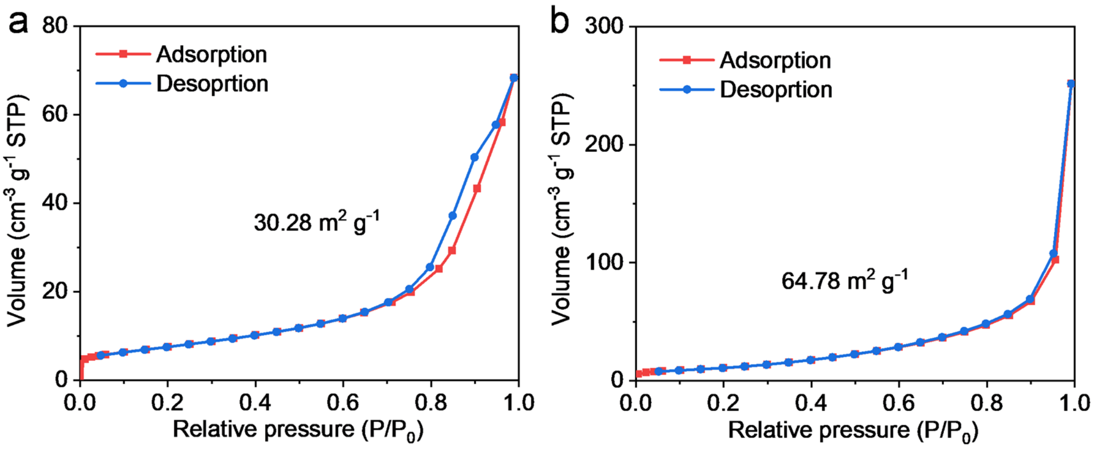

Supplementary Fig. 11| N_2 adsorption-desorption isotherms of (a) commercial RuO_2 and (b) Er-RuO_x.

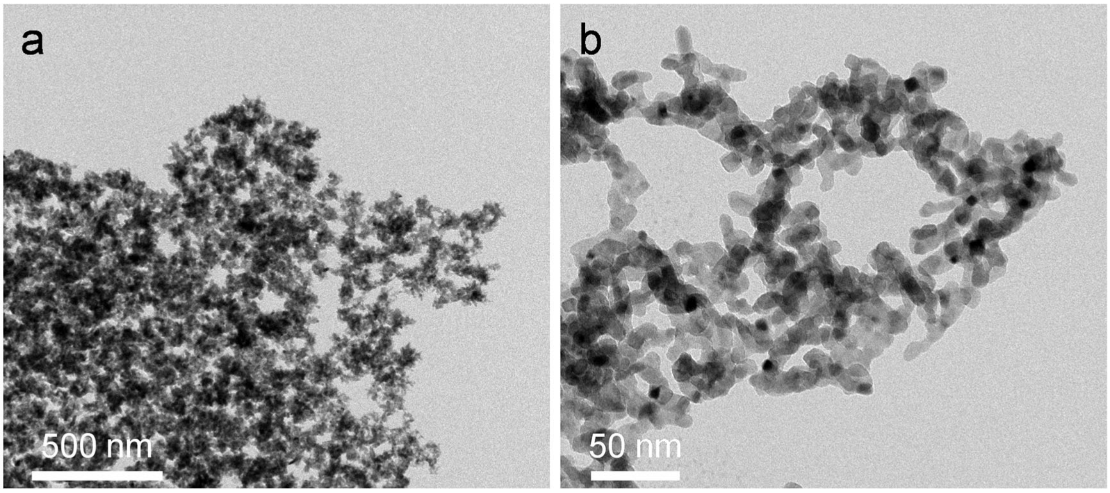

Supplementary Fig. 12| TEM characterization of Er-RuO_x. TEM images of Er-RuO_x at various magnification.

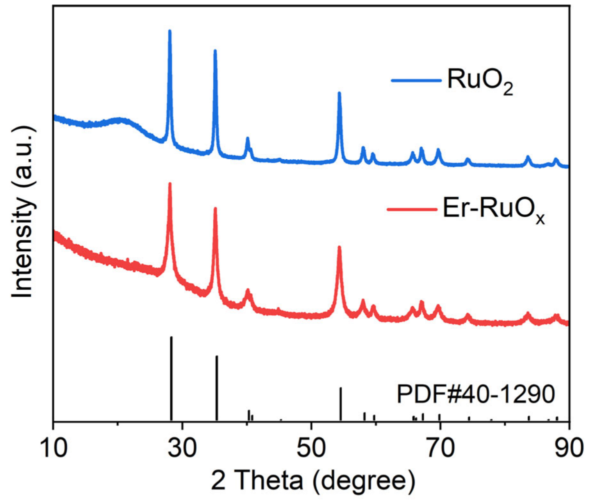

Supplementary Fig. 13| Phase characterization. XRD patterns of RuO_2 and Er-RuO_x.

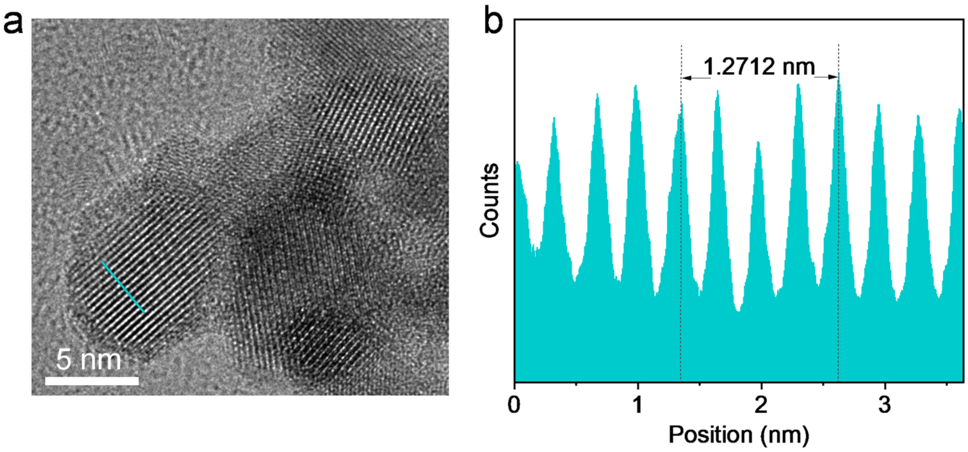

Supplementary Fig. 14| (a) HRTEM image and (b) linear analysis of Er-RuO_x.

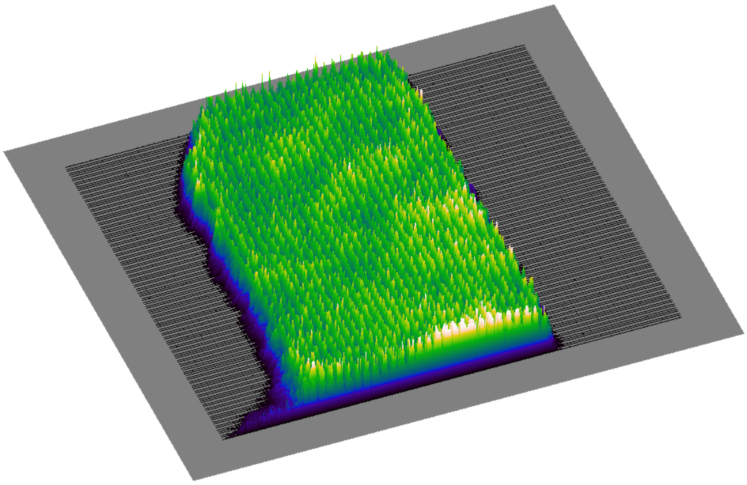

Supplementary Fig. 15| Surface plot of the Er-RuO_x along [110] zone axes.

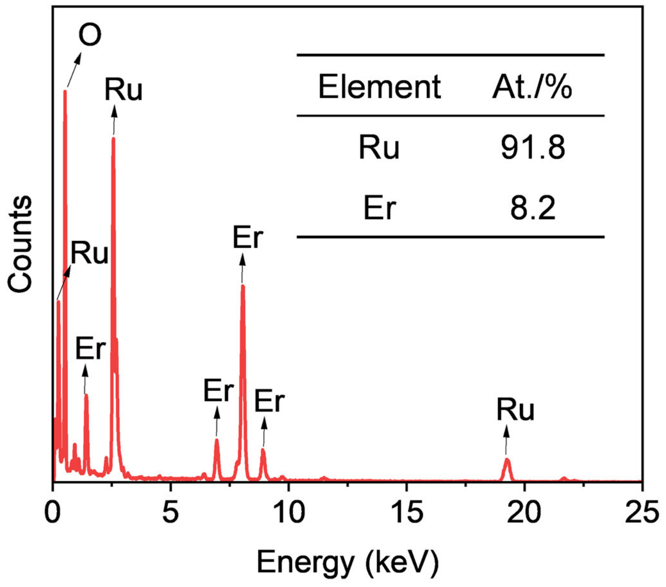

Supplementary Fig. 16| The EDS spectra of Er-RuO_x.

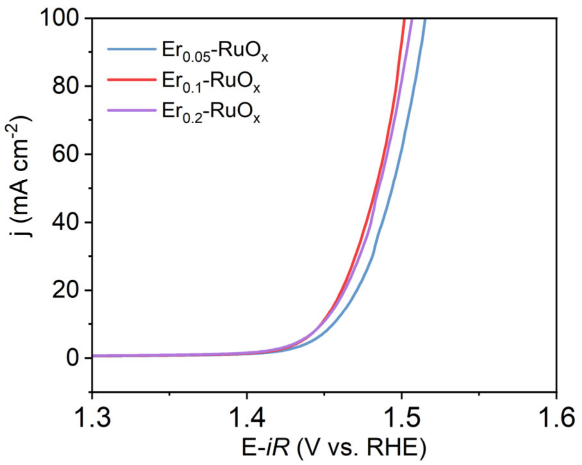

Supplementary Fig. 17| OER polarization curves of $Er_{0.05}$-RuO_x, $Er_{0.1}$-RuO_x and $Er_{0.2}$-RuO_x. Among them, $Er_{0.1}$-RuO_x exhibits the best OER activity. Unless otherwise specified, Er-RuO_x mentioned in the manuscript refers to $Er_{0.1}$-RuO_x.

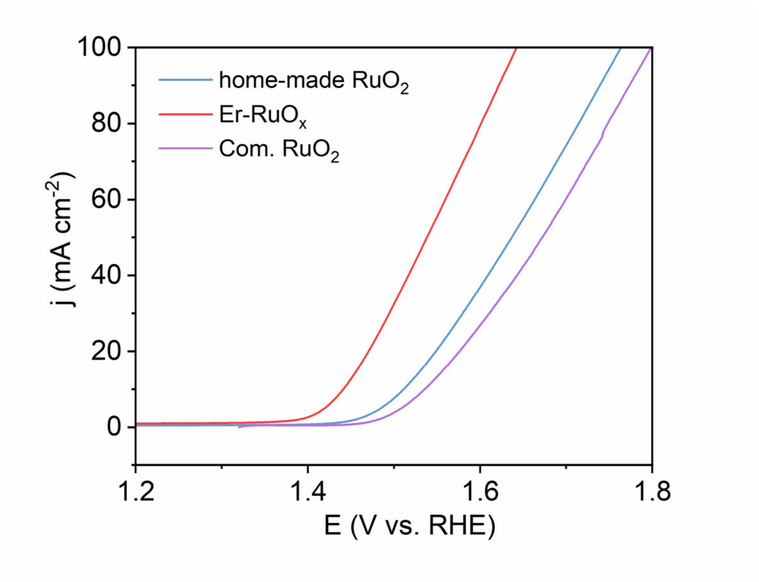

Supplementary Fig. 18| OER polarization curves of home-made RuO_2, Er-RuO_x and commercial RuO_2 without *iR* compensation.

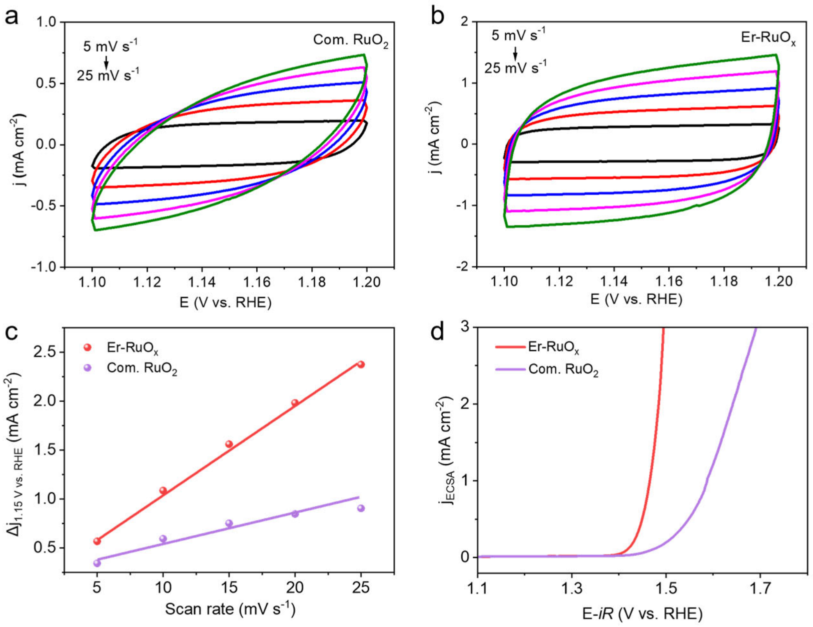

Supplementary Fig. 19| Electrocatalytic performance of the catalysts. Cyclic voltammetry curves of (a) commercial RuO_2 and (b) Er-RuO_x measured in the non-Faradaic region at different scan rates. (c) The current density against the scan rate of commercial RuO_2 and Er-RuO_x at 1.15 V. (d) Polarization curves normalized to electrochemical active area (ECSA).

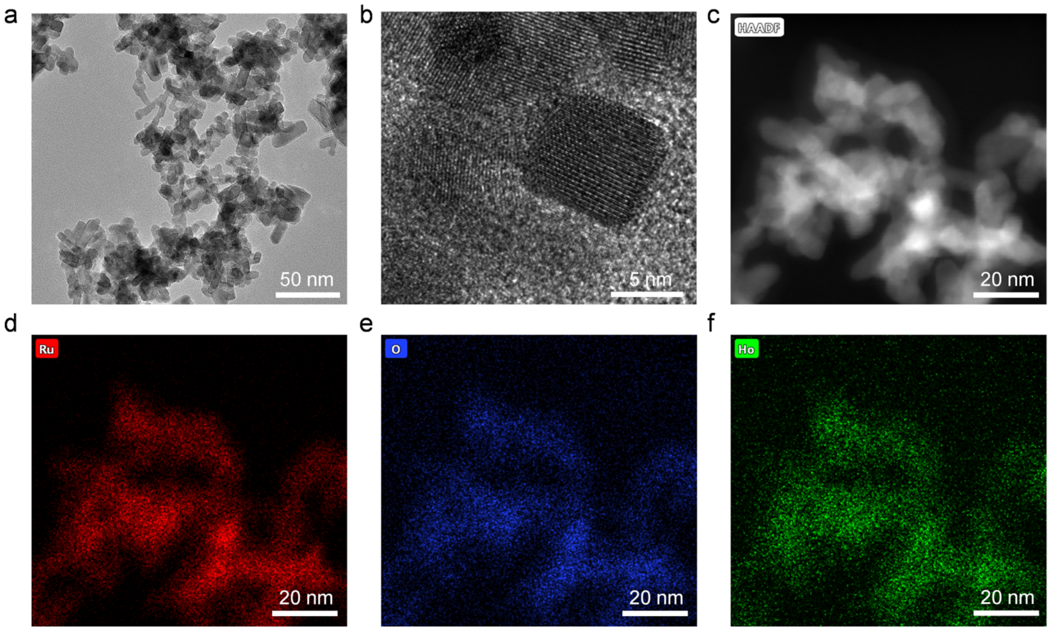

Supplementary Fig. 20| TEM characterization of Ho-RuO$_x$. (a) TEM and (b) HRTEM images of Ho-RuO$_x$. (c) HAADF-STEM image and (d-f) corresponding element mapping images of Ho-RuO$_x$.

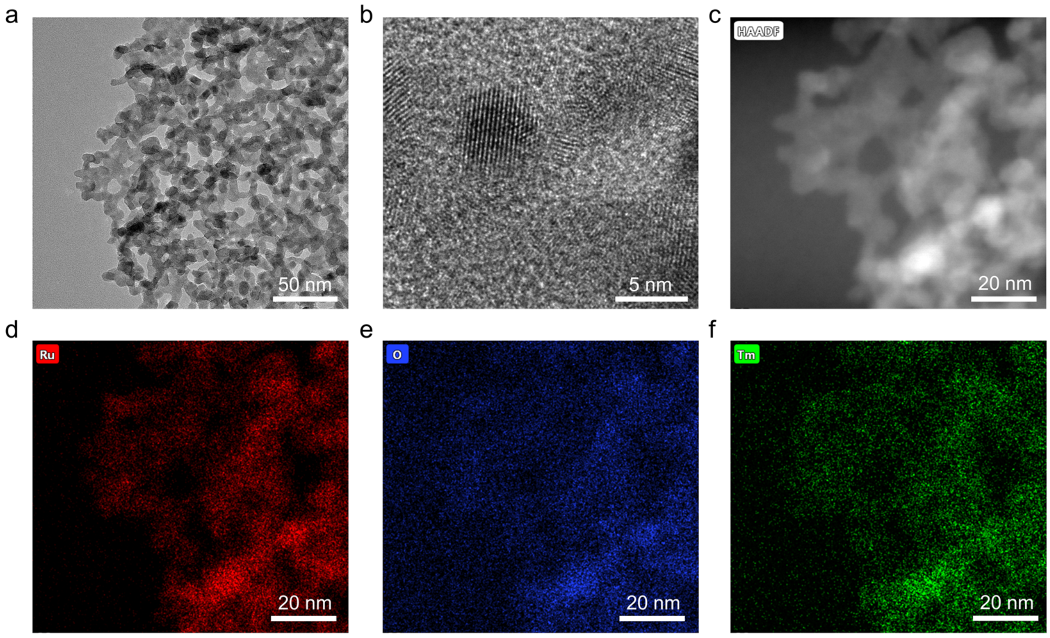

Supplementary Fig. 21| TEM characterization of Tm-RuO_x. (a) TEM and (b) HRTEM images of Tm-RuO_x. (c) HAADF-STEM image and (d-f) corresponding element mapping images of Tm-RuO_x.

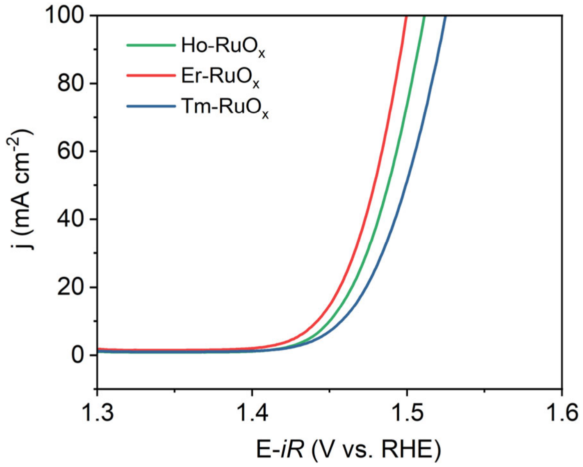

Supplementary Fig. 22| OER polarization curves of Ho-RuO_x, Er-RuO_x and Tm-RuO_x.

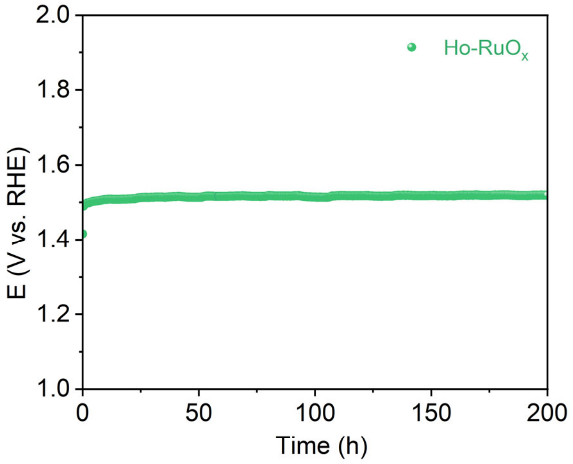

Supplementary Fig. 23| The CP curves of Ho-RuO$_x$ at 10 mA cm^{-2}.

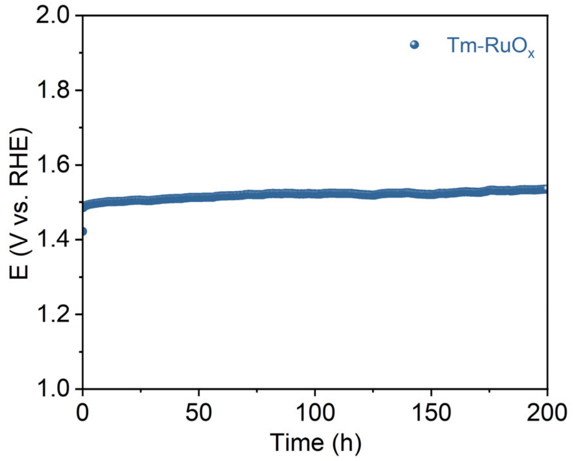

Supplementary Fig. 24| The CP curves of Tm-RuO$_x$ at 10 mA cm^{-2}.

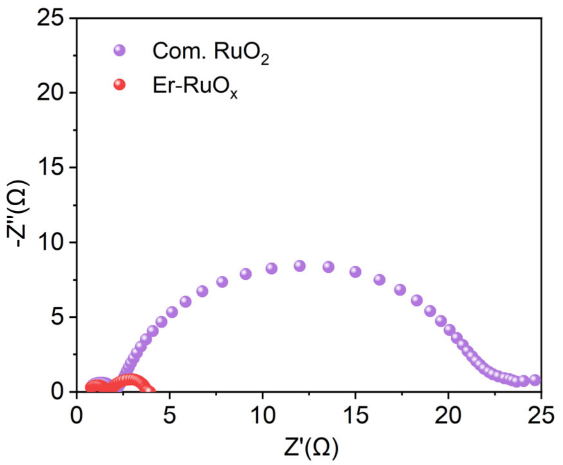

Supplementary Fig. 25| Nyquist plots of Er-RuO_x and commercial RuO_2 at 1.485 V vs. RHE.

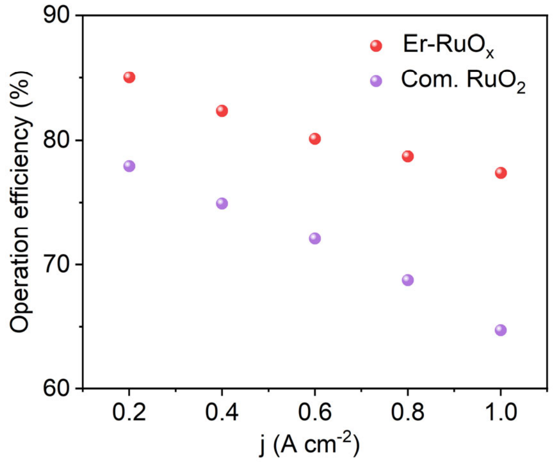

Supplementary Fig. 26| The PEMWE device efficiency using Er-RuO_x and commercial RuO_2 as the anode at 80 °C, respectively.

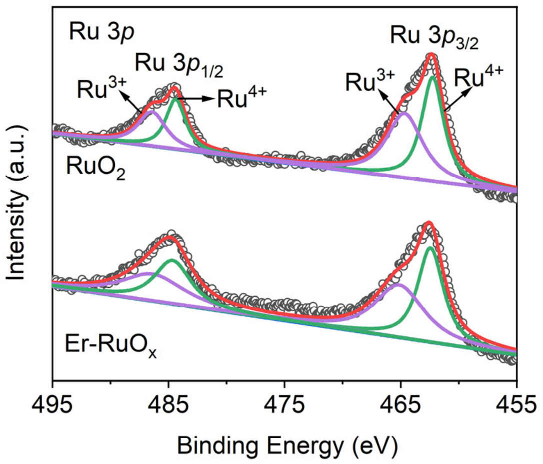

Supplementary Fig. 27| Ru 3*p* XPS patterns of Er-RuO_x and RuO_2.

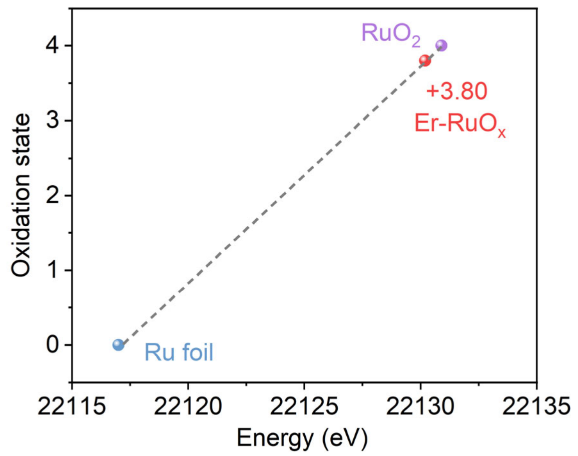

Supplementary Fig. 28| Oxidation state of Ru obtained from Ru K-edge XANES.

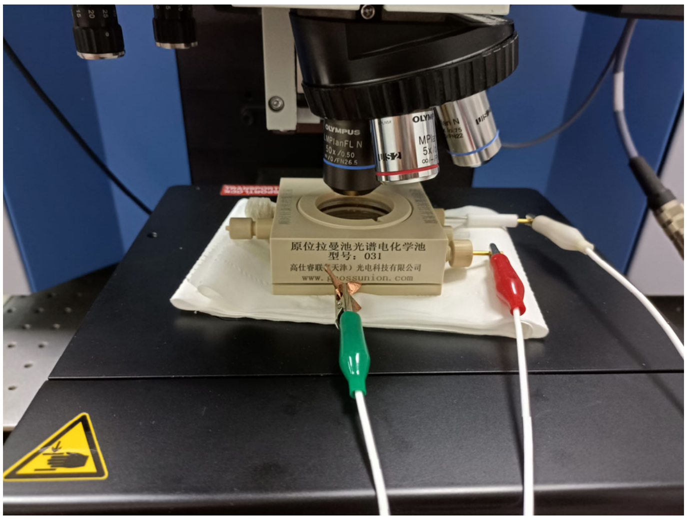

Supplementary Fig. 29| The photo of the in-situ Raman set-up.

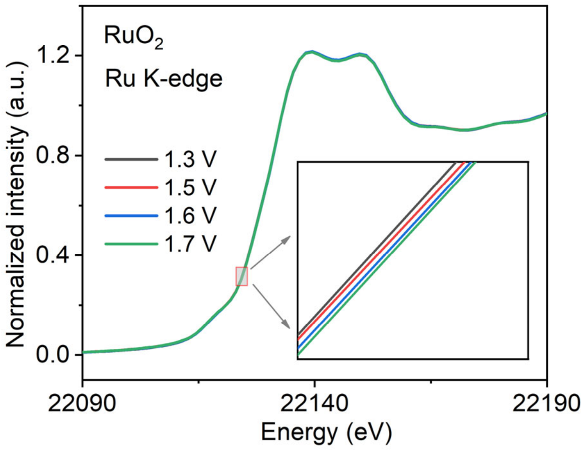

Supplementary Fig. 30| Ru K-edge XANES spectra of RuO_2 with applied bias.

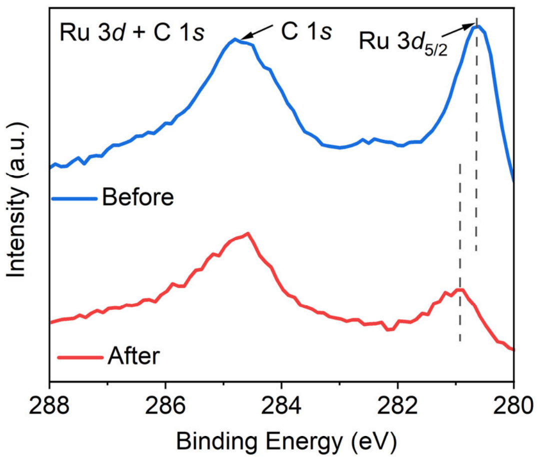

Supplementary Fig. 31| Ru 3*d* XPS patterns of Er-RuO_x before and after the stability test.

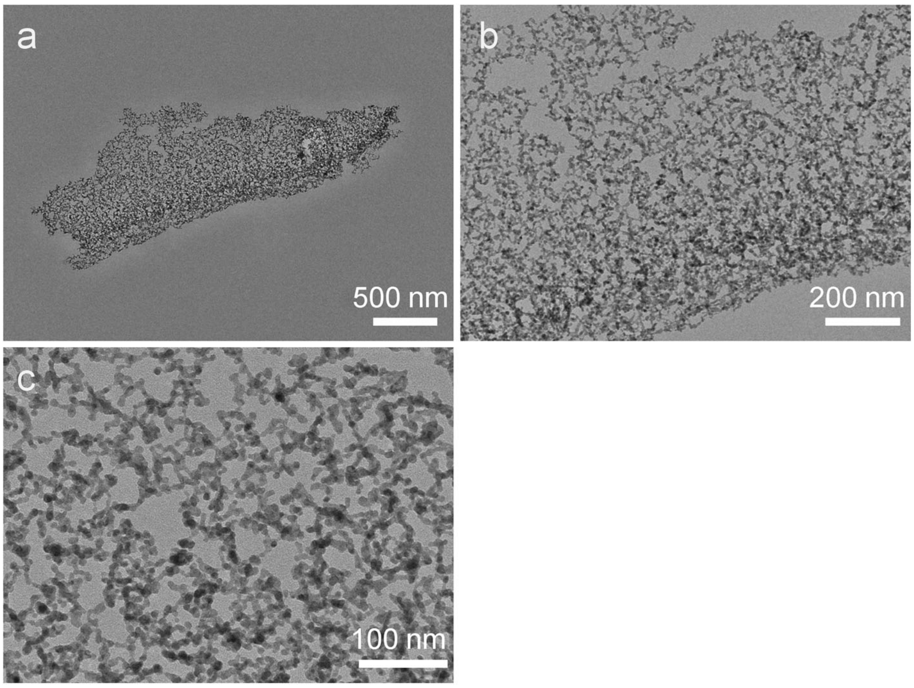

Supplementary Fig. 32| TEM images of Er-RuO_x at various magnifications after the stability test.

Supplementary Table 1| ΔE_{ZPE} and $T\Delta S$ for each OER intermediate on RuO_2.

Models	ΔE_{ZPE}-$T\Delta S$ (eV)
RuO_2-*OH	0.3859
RuO_2-*O	0.0935
RuO_2-*OOH	0.4043

Supplementary Table 2| ΔE_{ZPE} and $T\Delta S$ for each OER intermediate on Ho-RuO_x.

Models	ΔE_{ZPE}-$T\Delta S$ (eV)
Ho-RuO_x-*OH	0.4057
Ho-RuO_x-*O	0.1182
Ho-RuO_x-*OOH	0.355

Supplementary Table 3| ΔE_{ZPE} and $T\Delta S$ for each OER intermediate on Er-RuO_x.

Models	ΔE_{ZPE}-$T\Delta S$ (eV)
Er-RuO_x-*OH	0.3743
Er-RuO_x-*O	0.1367
Er-RuO_x-*OOH	0.3611

Supplementary Table 4| ΔE_{ZPE} and TΔS for each OER intermediate on Tm-RuO_x.

Models	ΔE_{ZPE}-TΔS (eV)
Tm-RuO_x-*OH	0.3912
Tm-RuO_x-*O	0.1224
Tm-RuO_x-*OOH	0.4031

Supplementary Table 5| The concentrations of dissolved Ln in Ln-RuO_x after 20-cycles accelerated durability test by ICP-MS.

Samples	Concentration (ug/L)
Ho-RuO_x	9.0877
Er-RuO_x	8.1903
Tm-RuO_x	10.1503

Supplementary Table 6| OER activity comparison of Er-RuO_x, commercial RuO_2 and those previously reported catalysts.

Catalysts	Substrate	Rotation	η @10 mA cm^{-2} /mV	Tafel / mv dec^{-1}	Loading amount	Electrolyte	Reference
Er-RuO_x	carbon paper	no	200	45	0.5 mg cm^{-2}	0.5 M H_2SO_4	This work
commercial RuO_2	carbon paper	no	287	105	1 mg cm^{-2}	0.5 M H_2SO_4	This work
Ni-RuO_2	glassy carbon electrode	2500 rpm	214	42.6	0.4 mg cm^{-2}	0.1 M $HClO_4$	Nat. Mater., 2023, 22(1), 100-108
py-RuO_2:Zn	Ti plate	no	173	41.2	0.52 mg_{Ru} cm^{-2}	0.5 M H_2SO_4	Nat. Commun., 2023, 14(1), 2517
Y_2MnRuO_7	rotating disk electrode	1600 rpm	270	47	0.25 mg cm^{-2}	0.1 M $HClO_4$	Nat. Commun., 2023, 14(1), 2010
Rh-RuO_2/G	glassy carbon electrode	/	161	45.8	0.45 mg cm^{-2}	0.5 M H_2SO_4	Nat. Commun., 2023, 14(1), 1412
$Nb_{0.1}Ru_{0.9}O_2$	rotation disc electrode	1600 rpm	204	47.9	0.51 mg cm^{-2}	0.5 M H_2SO_4	Joule, 2023, 7(3), 558-573
$SnRuO_x$	L shape gold electrode	/	194	38.2	41.65 µg cm^{-2}	0.5 M H_2SO_4	Nat Commun., 2023 14, 843.
RuAl	glassy carbon electrode	1600 rpm	192	48	0.3 mg cm^{-2}	0.5 M H_2SO_4	Chem, 2023, DOI: 10.1016/j.chempr.2023.08.006
$RuCoO_x$	carbon cloth	no	200	50.1	3.2428 mg cm^{-2}	0.1 M $HClO_4$	J. Am. Chem. Soc., 2023, 145(32), 17995-18006

Supplementary Table 7| The OER stability comparison of Er-RuO_x, commercial RuO_2 and those previously reported catalysts.

Catalysts	Substrate	Operation current density / mA cm^{-2}	Operation time / h	Degradation rate / μV h^{-1}	Electrolyte	Reference
Er-RuO_x	carbon paper	10	200	~160	0.5 M H_2SO_4	This work
Ni-RuO_2	rotation disc electrode	10	200	/	0.1 M $HClO_4$	Nat. Mater. 2023, 22(1), 100-108
Rh-RuO_2/G	Ti mesh	10	20	7995	0.5 M H_2SO_4	Nat. Commun., 2023, 14(1), 1412
PtCo-RuO_2/C	carbon paper	10	100	4470	0.1 M $HClO_4$	Energy Environ. Sci., 2022, 15(3), 1119-1130
Y_2MnRuO_7	/	10	40	564	0.1 M $HClO_4$	Nat. Commun., 2023, 14(1), 2010
py-RuO_2:Zn	Ti plate	50	1000	153	0.5 M H_2SO_4	Nat. Commun., 2023, 14(1), 2517
$RuCoO_x$	carbon cloth	10	100	450	0.1 M $HClO_4$	J. Am. Chem. Soc., *2023,* DOI: 10.1021/jacs.3c05556
RuAl	carbon paper	10	300	/	0.5 M H_2SO_4	Chem, 2023, https://doi.org/10.1016/j.chempr.2023.08.006
$Nb_{0.1}Ru_{0.9}O_2$	carbon paper	200	360	25	0.5 M H_2SO_4	Joule, 2023, 7(3), 558-573

Supplementary Table 8| PEMWE electrolyzer activity based on Er-RuO_x, commercial RuO_2 and those previously reported catalysts.

Anode catalysts	Loading amount	Membra-ne	Cell temperat-ure /°C	Cell voltage (V) at			Reference
				1 A cm^{-2}	2 A cm^{-2}	3 A cm^{-2}	
Er-RuO_x	3 mg cm^{-2}	Nafion 117	80 °C	1.590	1.713	1.837	This work
commercial RuO_2	3 mg cm^{-2}	Nafion 117	80 °C	1.901	/	/	This work
PtCo-RuO_2/C	2.5 mg cm^{-2}	Nafion 212	80 °C	1.621	1.736	1.872	Energy Environ. Sci., 2022, 15(3), 1119-1130
Y_2MnRuO_7	0.2 mg_{Ru} cm^{-2}	Nafion 212	80 °C	1.75	2.08	/	Nat. Commun., 2023, 14(1), 2010
$Nb_{0.1}Ru_{0.9}O_2$	2 mg cm^{-2}	Nafion 117	80 °C	1.69	1.91	2.13	Joule, 2023, 7(3), 558-573
Ni-RuO_2	3.1 mg cm^{-2}	Nafion 117	room temperat-ure	1.95	/	/	Nat. Mater., 2023, 22(1), 100-108
$Ru_{0.5}Ir_{0.5}O_2$	1 mg cm^{-2}	Nafion 117	/	~1.62	~1.82	/	Nat. Commun., 2023, 14(1), 5365
SnRuO_x	4 mg cm^{-2}	Nafion 212	80 °C	1.565	1.655	1.735	Nat. Commun., 2023 14(1), 843

Supplementary Table 9| EXAFS fitting parameters at the Ru K-edge（S_0^2=0.90*）

Sample	Path	C.N.	R (Å)	$\sigma^2 \times 10^3$ (Å^2)	ΔE (eV)	R factor
Ru foil	Ru-Ru	12*	2.68±0.01	3.9±0.3	-2.7±0.8	0.003
$Er\text{-}RuO_x$	Ru-O	1.8±0.2	1.98± 0.01	1.4±0.7	3.9±1.3	0.004
	Ru-Ru	2.1±0.6	3.14± 0.01	8.6±1.7	0.3±2.0	
	Ru-Ru	1.2±0.3	3.58± 0.01	1.3±1.2	9.1±2.0	
RuO_2	Ru-O	2.3±0.2	1.97± 0.01	2.5±0.8	3.8±1.4	0.006
	Ru-Ru	2.6±0.9	3.13± 0.01	7.7±2.2	0.1±2.3	
	Ru-Ru	1.2±0.4	3.58± 0.01	1.2±1.4	9.0±2.5	

C.N.: coordination numbers; *R*: bond distance; σ^2: Debye-Waller factors; Δ*E*: the inner potential correction; *R* factor: goodness of fit; * fitting with fixed parameter.

Supplementary Notes

Cost of per kilogram H_2[1]:

Cost (H_2/kg) = energy consumption × electricity bill

= 42.61 kW h/kg H_2 × $ 0.02/kW h

= $ 0.85/kg H_2

Calculation of oxidation states from XANES

Firstly, background deduction and normalization of χμ(E) data were executed before E_0 calibration with foil samples as standard references. Then E_0 of each studied sample were determined by their first derivative vertex (second peak for Ru element). Taking Ru foil and RuO_2 as references, the oxidation states of the samples with different E_0 values can be obtained through linear fitting.

Supplementary References

1. Hao, S. et al. Torsion strained iridium oxide for efficient acidic water oxidation in proton exchange membrane electrolyzers. *Nat. Nanotechnol*. **16**, 1371-1377 (2021).
