# Supplementary material for: Lanthanide-regulating Ru-O covalency optimizes acidic oxygen evolution electrocatalysis
Source: Nat Commun. 2024 Jun 11;15:4974. doi: 10.1038/s41467-024-49281-2 (PMC11166638; doi:10.1038/s41467-024-49281-2)
Supplement: Supplementary file 3 — Description of Additional Supplementary Files [file 41467_2024_49281_MOESM3_ESM.pdf]

Description of Additional Supplementary Files

Supplementary Data 1: The optimized structure of Ho-RuOx
